# Mortality among Fire Department of the City of New York Rescue and Recovery Workers Exposed to the World Trade Center Disaster, 2001–2017

**DOI:** 10.3390/ijerph17176266

**Published:** 2020-08-28

**Authors:** Hilary L. Colbeth, Rachel Zeig-Owens, Charles B. Hall, Mayris P. Webber, Theresa M. Schwartz, David J. Prezant

**Affiliations:** 1 Fire Department of the City of New York, Bureau of Health Services, 9 Metrotech Center, Brooklyn, NY 11201, USA; Hilary.Colbeth@fdny.nyc.gov (H.L.C.); Mayris.Webber@fdny.nyc.gov (M.P.W.); Theresa.Schwartz@fdny.nyc.gov (T.M.S.); David.Prezant@fdny.nyc.gov (D.J.P.); 2 Department of Medicine, Pulmonology Division, Montefiore Medical Center, Bronx, NY 10467, USA; 3 Department of Epidemiology and Population Health, Albert Einstein College of Medicine, Bronx, NY 10461, USA; charles.hall@einsteinmed.edu; 4 Department of Medicine, Pulmonology Division, Albert Einstein College of Medicine, Bronx, NY 10461, USA

**Keywords:** mortality, disaster epidemiology, world trade center, rescue/recovery workers, occupational exposure

## Abstract

The World Trade Center (WTC) attacks on 11 September 2001 have consistently been associated with elevated rates of physical and mental health morbidities, while evidence about mortality has been limited. We examined mortality between 12 September 2001 and 31 December 2017 among 15,431 WTC-exposed Fire Department of the City of New York (FDNY) firefighters and emergency medical service providers (EMS), specifically assessing associations between intensity of WTC-exposure and mortality risk. Standardized mortality ratios (SMR) and 95% confidence intervals (CI) compared FDNY cohort mortality with the US general population using life table analysis. Deaths were identified via linkage to the National Death Index. Cox proportional hazards regression models were used to identify associations between intensity of WTC exposure and mortality, accounting for age, sex, race/ethnicity, smoking history, and other relevant confounders. We identified 546 deaths and a lower than expected all-cause mortality rate (SMR = 0.34; 95% CI, 0.31–0.37). No cause-specific SMRs were meaningfully elevated. Mortality hazard ratios showed no association or linear trend with level of WTC exposure. Our results provide evidence of the healthy worker effect, despite exposure to the World Trade Center. More follow-up time may be needed to assess the full impact of WTC exposure on mortality in this occupational population.

## 1. Introduction

The collapse of the World Trade Center (WTC) buildings after the September 11, 2001 terrorist attacks exposed Fire Department of the City of New York (FDNY) rescue/recovery workers to caustic dust and products of combustion [1]. The disaster’s intense dust cloud contained a huge variety of irritants, including partially combusted and/or pulverized wood, paper, and jet fuel; construction materials including asbestos, glass, silica, fiberglass, and concrete; complex organic chemicals; lead and other metals [1,2]. Subsequently, several physical and mental health conditions were linked to 9/11/2001-related exposures, such as high rates of airway injury including excessive loss of lung function [3], excess cancer risks [4,5,6], aerodigestive illnesses or gastroesophageal reflux disease (GERD) [7], long-term cardiovascular disease risk [8] and increased risks of post-traumatic stress disorder (PTSD) [9], depression [10], and binge drinking [11,12] among rescue/recovery workers and area residents. Substantial comorbidities between these health conditions have also been documented [13,14,15].

While some previous studies investigated whether these conditions led to an excess in mortality [16,17,18], to date, none have found a consistently significant association with WTC-exposure and none have been specific to FDNY WTC-exposed rescue/recovery workers: firefighters and emergency medical service providers (EMS). An early study from the WTC Health Registry cohort did not report an association between exposure and mortality (2003–2009) among rescue/recovery workers [16]. However, the 2018 follow-up investigation, using an additional five years of observation (2003–2014), found that despite a significantly lower than expected all-cause mortality among rescue/recovery workers and community members (standardized mortality ratios [SMR] = 0.69; 0.86, respectively), rescue/recovery workers with intermediate or high levels of WTC-exposure experienced an increased risk of all-cause mortality compared with those with lower levels of WTC-exposure (intermediate and high exposure adjusted hazard ratios [AHR] = 1.36; 1.41, respectively) [17]. In addition, some specific causes of death, such as suicide among rescue/recovery workers (SMR = 1.82), were significantly elevated. Using National Death Index (NDI) data between 2002 and 2011, the WTC Health Program General Responder Cohort studied mortality rates in their rescue/recovery worker cohort and found that all-cause mortality was not elevated and that there was no association between mortality and WTC-exposure intensity or duration [18].

Given the influence of the healthy worker effect among those who participated in the rescue/recovery and the long latency periods and/or median survival times [19,20] of many WTC-related conditions, previous studies may not have had sufficient follow-up time to detect excess mortality. Furthermore, those enrolled in the WTC Health Program are likely to have been screened during routine medical monitoring and treatment visits at levels well above the general population [7]. Early disease identification and treatment in this population may have improved survival.

The current study assessed the relationship between WTC-exposure and mortality risk among FDNY firefighters and EMS. This work built upon and extended previous WTC-related mortality studies in other cohorts [16,17,18] by concentrating on a more homogenous population created prior to 9/11/2001, that had relatively higher WTC-exposures, lower longitudinal dropout, and longer years of follow-up.

## 2. Materials and Methods

### 2.1. Study Population

The source population consisted of 15,478 FDNY firefighters and EMS who arrived to work at the WTC-site between the morning of 9/11/2001 and 7/25/2002. Rescue/recovery workers who were actively employed by FDNY for less than 18-months were excluded (*n* = 47) to ensure that all included participants were actively employed as a firefighter or EMS after completing training [4]. The final study population consisted of 15,431 participants.

Demographic data were obtained from the FDNY employee database. Information on WTC-site arrival time and smoking status was derived from self-administered questionnaires completed during routine medical monitoring examinations at FDNY. Participants of unknown race (*n* = 46, 0.3%) were assumed to be non-Hispanic white, because all were hired prior to 1983 when the majority of FDNY was non-Hispanic white. Level of WTC exposure was defined based on the FDNY-WTC exposure intensity index [21] as being high (arrived on the morning of 9/11/2001), moderate (arrived afternoon of 9/11/2001 or on 9/12/2001), or low (arrived between 9/13/2001 and 07/25/2002).

### 2.2. Ascertainment of Deaths

We provided identifying FDNY cohort information (first/last names, date of birth, sex, race, marital status, social security number [SSN], and state of residence) to the National Death Index (NDI), a centralized database of death records from state-based vital statistics offices [22]. NDI identified all deaths in this cohort that occurred from 9/12/2001 through 12/31/2017. The sensitivity of the NDI is over 95% when SSN are available [23]; the FDNY provided SSN for all study records. Underlying causes of death were coded conforming to the International Classification of Disease codes, 10th revision (ICD-10) [24].

### 2.3. Statistical Analysis

Demographic and other characteristics of the study population were assessed as proportions, medians, interquartile ranges, and means (SDs), as appropriate. The observation period began on 12 September 2001 and follow-up ended at death or the study end date (31 December 2017), whichever was earlier. The National Institute for Occupational Safety (NIOSH) Life Table Analysis System (LTAS.NET) [25,26] was used to calculate standardized mortality ratios with the US general population as the referent (1960–2019), and RStudio version 4.1 was used to check the LTAS output. The underlying cause of death was categorized per LTAS’s 119-cause rate file [25] which uses ICD-10 codes mapped to 119 minor and 28 major cause of death categories [24]. For each rescue/recovery worker, observed person-years-at-risk were stratified by sex (male, female), race (non-Hispanic white, non-white, non-Hispanic black, Hispanic, and other), and five-year bands of age and calendar period. Yearly expected mortality was calculated by multiplying the stratum’s person-years-at-risk by the corresponding cause-specific US population mortality rate [25]. The expected numbers were summed across strata to obtain cause-specific and total expected number of deaths. SMRs were estimated as the ratio of the observed to the expected deaths. Ninety-five percent confidence intervals (CI) were estimated based on a Poisson distribution for the observed outcome, with exact limits for outcomes with 10 or fewer occurrences [27].

All-cause and cause-specific SMRs were examined overall and stratified by race (non-Hispanic white, non-white), work assignment (firefighters, EMS), and WTC-exposure (high, moderate/low). All LTAS major causes of death were reported and minor causes of death were chosen a priori based on the results of previous studies [17,28] and knowledge of the cohort. Minor causes of death were: human immunodeficiency virus (HIV), malignant neoplasms of the esophagus, pancreas, trachea/bronchus/lung, melanoma, mesothelioma, and the brain and other parts of the nervous system; non-Hodgkin’s lymphoma, multiple myeloma, benign and unspecified neoplasms of the eye, brain, and other parts of the nervous system, alcoholism, other nervous system diseases, rheumatic heart disease, hypertension with heart disease, ischemic heart disease, cerebrovascular disease, hypertension without heart disease, diseases of the arteries/veins/lymph nodes, pneumonia, chronic obstructive pulmonary disease (COPD), hernia and intestinal obstruction, chronic and unspecified renal failure, motor vehicle (-driver, -pedestrian), fire in a building, accidental poisoning, intentional self-harm (hereafter, this LTAS category (minor cause 116) referred to as “suicide”), and assault and homicide.

To examine the association between level of WTC-exposure and mortality, we conducted a secondary analysis using multivariable Cox proportional hazards regression. We fit unadjusted and adjusted models using WTC-exposure (high or moderate/low) to predict the risk of all-cause, cancer, heart disease, and respiratory disease mortality. Adjusted models were then stratified by race (non-Hispanic white vs. non-white); however, not by sex, due to small sample size. All models used time since 9/11/2001 as the time scale and the adjusted models included age on 9/11/2001, sex (male or female), race (non-Hispanic white or non-white), work assignment (firefighter or EMS), smoking status (ever or never), and body mass index (BMI) at the end of follow-up as covariates. Respiratory disease mortality models were restricted to non-Hispanic whites and were not stratified by race, as all respiratory disease-related deaths (*n* = 28) occurred among non-Hispanic whites. To assess a WTC-exposure gradient within the adjusted models, a test for trend was conducted using the high, moderate, and low WTC-exposure categories. SAS software version 9.4 (SAS Institute Inc., Cary, NC, 27513, USA) was used for demographic and cox proportional hazards analyses.

## 3. Results

### 3.1. Characteristics of the Study Sample

Characteristics of the final study population are presented in Table 1. The median age on 9/11/2001 of rescue/recovery workers was 39.9 (interquartile range (IQR), 33.6–46.2). Participants were largely male (96.8%), firefighters (86.0%), non-Hispanic white (87.2%), never-smokers (60.9%), and arrived to work at the WTC during the afternoon of 9/11/2001 or 9/12/2001 (63.6%). BMI at the end of follow-up for the total population ranged from 15.6–65.2 with a mean of 30.5 kg/m^2^; only 2% of the population were underweight (BMI < 18.5 kg/m ^2^).

From 9/12/2001 to 12/31/2017, we identified 546 deaths among 15,431 FDNY rescue/recovery workers, during a total of 248,664.5 person-years of observation. The median age at death was 58.9 (IQR, 51.6–68.5) years, and the median time from the start of follow-up to death was 11.6 (IQR, 8.7–14.0) years. Those who died were similar to the overall study population in the distributions of sex, race, mean BMI, and work assignment, but were older on 9/11/2001, more likely to report ever-smoking and had a slightly higher percentage of individuals who arrived to the WTC site after 9/12/2001 (Table 1). The most common causes of death were cancer (*n* = 197) and heart disease (*n* = 113).

### 3.2. Mortality Among the Full Cohort

Compared to the US population referent, all-cause mortality was significantly lower than expected (SMR = 0.34; 95% CI, 0.31–0.37) (Table 2). Similarly, SMRs for many of the major cause-of-death categories, including all cancers (SMR = 0.46; 95% CI, 0.40–0.54), diseases of the heart (SMR = 0.33; 95% CI, 0.27–0.40), other diseases of the circulatory system (SMR = 0.25; 95% CI, 0.16–0.38), and diseases of the respiratory system (SMR = 0.27; 95% CI, 0.18–0.39), were significantly lower than expected. Among cancer deaths, those with the most observed cases demonstrated significantly lower than expected mortality: digestive organs and peritoneum (SMR = 0.56; 95% CI, 0.43–0.70), respiratory system (SMR = 0.28; 95% CI, 0.20–0.39), and other and unspecified sites (SMR = 0.49; 95% CI, 0.34–0.69). Observed deaths and SMRs among NIOSH minor cause of death categories, such as suicide, are presented in Table 3. None were significantly elevated in the overall population, except for mortality caused by building fires (SMR = 3.72; 95% CI, 1.70–7.06).

### 3.3. Race, Work Assignment, and WTC-Exposure

All-cause mortality among non-Hispanic whites (SMR = 0.33; 95% CI, 0.30–0.36) and non-whites (SMR = 0.44; 95% CI, 0.35–0.55) was similar and significantly below the US general population (Table 4). Cancers were the leading cause of death among non-Hispanic whites and non-whites (SMR = 0.44; 95% CI, 0.38–0.52, n = 164 and SMR = 0.66; 95% CI, 0.44–0.96, n = 27), resulting in approximately 35% of deaths in each respective group. Cancer deaths among non-whites were closely followed in number by heart disease mortality (SMR = 0.53; 95% CI, 0.33–0.81; n = 22). All respiratory disease deaths occurred among non-Hispanic whites (SMR = 0.29; 95% CI, 0.19–0.42; n = 28).

Table 5 shows that firefighters were characterized by decreased all-cause mortality (SMR = 0.31; 95% CI, 0.29–0.34), all-cancer mortality (SMR = 0.43; 95% CI, 0.37–0.51), heart disease mortality (SMR = 0.29; 95% CI, 0.24–0.36) and respiratory disease mortality (SMR = 0.27; 95% CI, 0.17–0.39). Firefighters also showed significant decreases compared with the US general population from mental-health-related causes of death, such as suicide (SMR = 0.31; 95% CI, 0.19–0.49; n = 19) and alcoholism (SMR = 0.33; 95% CI, 0.11–0.77; n ≤ 5) (data not shown). Only death from building fires showed an excess (SMR = 4.26; 95% CI, 1.94–8.09; n = 9) (data not shown); all of these deaths occurred in firefighters who were on active duty (i.e., during work). Among EMS, results in major cause of death categories were similar to those of firefighters.

Those who arrived the morning of 11 September 2001 (high WTC exposure) demonstrated significantly lower than expected all-cause mortality (SMR = 0.41; 95% CI, 0.33–0.51), all-cancer mortality (SMR = 0.51; 95% CI, 0.33–0.74), and heart disease mortality (SMR = 0.30; 95% CI, 0.16–0.51) (Table 6). No major causes of death were significantly elevated. Results were similar among those with moderate/low WTC exposure (arrived the afternoon of 11 September 2001–25 July 2002).

### 3.4. Associations of WTC-exposure, Smoking History and Mortality

We examined factors associated with all-cause, cancer, heart, and respiratory disease-related deaths separately, by level of WTC exposure. In multivariable models (Table 7), arrival to the WTC-site the morning of 9/11/2001 did not demonstrate a significantly elevated hazard of mortality from all-causes (AHR = 1.24; 95% CI, 0.98–1.58), cancer mortality (AHR = 1.15; 95% CI, 0.75–1.78), heart disease mortality (AHR = 0.93; 95% CI, 0.52–1.68), or respiratory mortality (AHR = 1.44; 95% CI, 0.49–4.23). Ever smoking predicted the highest risk for all-cause (AHR = 1.54, 95% CI, 1.27–1.86), cancer (AHR = 1.73, 95% CI, 1.24–2.42), and heart disease (AHR = 1.89, 95% CI, 1.22–2.93) mortality. Stratified by race, AHR results for all-cause, cancer, and heart disease mortality among non-Hispanic whites and non-whites were similar (data not shown). When assessing WTC-exposure as a dose-response variable predicting the hazard of mortality from all causes, cancer, heart, and respiratory disease, trends were not statistically significant (*p*-value = 0.79, 0.79, 0.48, 0.82, respectively) (data not shown).

## 4. Discussion

With nearly 17 years of follow-up, all-cause and a majority of cause-specific mortality rates among WTC-exposed FDNY rescue/recovery workers were lower than expected in comparison with the US general population. The lack of differences between SMRs stratified by race, work assignment (firefighter vs. EMS), and WTC-exposure level suggest that the overall study findings are robust. In regard to cancer, cardiovascular, and respiratory deaths, FDNY rates remained far lower than those of the US general population. Although the association between WTC exposure and increased risk of mortality from certain diseases may be explored by more targeted research, our results, stratified by level of exposure, do not suggest an association; however, we acknowledge potential confounding by the healthy worker effect [29,30,31]. These findings are also supported by our Cox proportional hazards analyses and are consistent with patterns reported by other mortality investigations among WTC-exposed cohorts [16,17,18].

It is possible that the use of SMRs resulted in an attenuation of effect estimates. In general, SMRs likely underestimate true relative risks, since they rely on the general population as the unexposed reference group-one which is not entirely comparable to the FDNY cohort in terms of underlying health status, since it includes individuals unfit for work. Working populations typically experience a 20% deficit in overall mortality rates below the general population, often ascribed to the healthy worker effect [32,33]. Additionally, there are health requirements for becoming a firefighter or EMS [34] and the SMR is often affected by the healthy worker hire effect, as people who are hired into physically demanding jobs are healthier at baseline than the general population [35]. This selection bias into employment is magnified by the requirement that firefighters and EMS must pass annual medical evaluations throughout their career. Thus, SMRs and the healthy worker effect may partially mask the harmful effects of occupational exposures, such as the WTC. Furthermore, the impact of the healthy worker effect in the FDNY population may not allow our results to be generalizable to other WTC-exposed cohorts. While we found lower mortality than a study of non-WTC-exposed US firefighters, we suspect that some of the differences in SMRs from those of the FDNY may be explained, in part, by the earlier average year of hire and longer follow-up time since hire of the non-WTC-exposed US firefighters [28]. This indicates an older population that is farther from the point of workforce entry and therefore, likely to have experienced a diminution of the healthy worker effect, bringing their mortality rates closer to that of the US general population. However, potential bias in FDNY results from the healthy worker effect can be reduced by controlling for variables such as job category and by using internal populations as a comparison [30,36,37]. Stratifying the results of our SMR analyses by work assignment, did not elucidate differences in mortality and our Cox proportional hazards models demonstrated no clear association between WTC exposure level and deaths occurring between 12 September 2001 and the end of 2017.

With some exceptions, our results are consistent with SMRs previously reported by the WTC Health Registry and the WTC Health Program General Responder Cohort. Our all-cause mortality ratio (SMR = 0.34; 95% CI, 0.31–0.37) was lower than both the rescue/recovery workers of the WTC Health Registry [17] (SMR = 0.69, 95% CI, 0.65–0.74) and the General Responder Cohort [18] (SMR = 0.43; 95% CI, 0.39–0.48). In contrast with the WTC Health Registry rescue/recovery workers, the FDNY cohort did not experience higher than expected mortality rates, even among the minor cause categories which included malignant cancers, such as pancreatic or non-Hodgkin’s lymphoma, nervous system diseases, and suicide. In addition, our Cox proportional hazards model results differed from the WTC Health Registry, which found significant associations between higher levels of WTC exposure and all-cause mortality among rescue/recovery workers. These differences may be partially explained by the WTC Health Registry rescue/recovery cohort being composed mostly of police, construction, and communication workers who did not receive pre-hire and annual medical exams prior to 11 September 2001 and therefore may not be influenced by the healthy worker effect to the same degree as the FDNY cohort [17]. Regarding the WTC-exposed General Responder Cohort, FDNY cause-specific mortality results were similar, with the exception of neoplasms of lymphatic and hematopoietic tissue and benign and unspecified neoplasms, which they found to be slightly elevated. The General Responder Cohort also reflected the results of our Cox proportional hazards model in their report of an increased, albeit non-significant, risk of all-cause mortality among those directly in the dust cloud (AHR = 1.13; 95% CI, 0.81–1.59), an equivalent measure to our high exposure category, and likewise did not identify an exposure-response gradient (p-trend = 0.88).

Lower than expected mortality rates are not surprising given that both WTC-exposed FDNY and General Responder rescue/recovery worker cohorts receive no-cost (including no co-pays or deductibles) physical and mental health annual monitoring examinations, screening tests, diagnostic procedures and treatments, and disease management counseling as part of the WTC Health Program. At FDNY, not only are these benefits offered by the WTC Health Program, but the program’s pre-existing health infrastructure developed credibility with the workforce prior to, during, and after the 11 September 2001 attacks, all of which may have facilitated member participation in testing and treatment and, consequently, improved survival. 

Expected associations for well-established risk factors of premature mortality in our cohort provide confidence in our results. For example, SMRs of the cause-specific minor categories were overwhelmingly lower than expected compared with the general population, with the exception of elevated rates of death from a building fire (SMR = 3.72), which is not surprising given that our population was nearly 89% firefighters, and, though not significantly, mesothelioma (SMR = 2.05), which has been reported as elevated among firefighters compared with the general population [28,38]. Secondly, as was emphasized through SMR stratification by WTC exposure, our Cox proportional hazards analyses demonstrated that WTC exposure intensity was not related to mortality risk while more typical risk factors such as being male, cigarette smoking, and older age conferred increased risk. Of the known correlates, the strongest association was with smoking history, in which ever-smokers demonstrated 50% or greater increased risk of death from all-causes, cancer, and heart disease compared with never-smokers. Ever smoking was not associated with respiratory disease mortality, likely due to a lack of statistical power with only 28 deaths due to respiratory disease.

Limitations of our study, in addition to the healthy worker effect, included a lack of information about certain lifestyle factors for the reference population, such as dietary intake, which some studies suggest differs between those in the fire service and the general population [39,40,41,42]. Our cohort also contained relatively few women and non-white rescue/recovery workers compared with the general population. Another limitation was that all deaths from 12 September 2001 to 31 December 2017 were included, regardless of the timing of the illness onset that ultimately contributed to these deaths. Thus, some deaths due to illnesses with long median survival times, including cancer, may have resulted from disease processes that began before the WTC disaster.

This study has several strengths. First, it was based on data from a large, longitudinally tracked cohort of WTC-exposed rescue/recovery workers formed prior to the 11 September 2001 exposure and included an observation period that extended three years longer than previous WTC mortality studies. In addition, SSN was available for 100% of participants in our cohort, leading to a nearly complete NDI match. Second, our study used well-established methods to ascertain the impact of WTC exposure on mortality and validated those results in a secondary analysis. Finally, this research is consistent with the findings of several other WTC-exposed mortality studies and establishes the current risk to the FDNY cohort, which can be referenced over time as follow-up is extended.

## 5. Conclusions

After a follow-up of nearly two decades, all-cause and cause-specific mortality ratios among FDNY rescue/recovery workers exposed to the WTC disaster were not elevated. Further, we did not demonstrate an association between level of WTC-exposure and mortality. Lower mortality in a cohort of workers healthy at baseline is generally expected; however, it is uncertain whether these outcomes will endure with additional years of follow-up. As the no-cost annual monitoring and treatment provided by the WTC Health Program may have contributed to the lower than expected mortality rates, we believe it to be critical that WTC-exposed rescue/recovery workers continue their full participation in the program.

## Figures and Tables

**Table 1:** Study population characteristics

Characteristic	Total Population	Deaths
	*N* (%)	*N* (%)
Total	15,431 (100)	546 (100)
Person-years of follow-up	248,664.50	5977.9 (100)
Age on 9/11/2001, median (IQR)	39.9 (33.6–46.2)	47.7 (41.9–57.1)
BMI (kg/m^2^) at end of follow-up, mean (SD) ^1^	30.5 (5.0)	30.2 (6.2)
Year hired, mean (SD)	1988 (10)	1980 (12)
WTC exposure Level		
High	2370 (15.4)	86 (15.8)
Moderate	9808 (63.6)	294 (53.9)
Low	3253 (21.1)	166 (30.4)
Race/ethnicity		
non-Hispanic white	13,456 (87.2)	468 (85.7)
Non-white ^2^	1975 (12.8)	78 (14.3)
Work assignment		
Firefighter	13,266 (86.0)	455 (83.3)
Emergency medical service providers	2165 (14.0)	91 (16.7)
Sex		
Male	14,943 (96.8)	532 (97.4)
Female	488 (3.2)	14 (2.6)
Smoking status		
Never smoker	9397 (60.9)	215 (39.4)
Ever smoker	5742 (37.2)	277 (50.7)
Unknown	292 (1.9)	54 (9.9)
Age at death, median (IQR)	N/A	58.9 (51.6–68.5)
Years to death post-9/11/2001, median (IQR)	N/A	11.6 (8.7–14.0)

Abbreviations: interquartile range (IQR), body mass index (BMI), World Trade Center (WTC). ^1^ Body mass index data only available for *n* = 15,072 in the total population and *n* = 484 among the deceased. ^2^ Non-Hispanic black, Hispanic, other.

**Table 2:** Observed deaths and standardized mortality ratios (SMRs) for major cause of death categories among World Trade Center-exposed Fire Department rescue/recovery workers of the City of New York, 12 September 2001–31 December 2017, compared with US population rates ^1.^

Cause (National Institute for Occupational Safety (NIOSH) Major Category) ^2^	Observed	SMR (95% CI)
All causes	546	0.34 (0.31–0.37) **
Tuberculosis and HIV related disease (01)	≤5	0.15 (0.03–0.44) **
All cancers	191	0.46 (0.40–0.54) **
MN buccal and pharynx (02)	≤5	0.18 (0.02–0.65) **
MN digestive organs and peritoneum (03)	70	0.56 (0.43–0.70) **
MN respiratory system (04)	35	0.28 (0.20–0.39) **
MN breast (05)	≤5	0.45 (0.01–2.51)
MN female genital organs (06)	≤5	1.09 (0.01–6.05)
MN male genital organs (07)	7	0.36 (0.14–0.74) **
MN urinary (08)	11	0.47 (0.23–0.84) **
MN other and unspecified sites (09)	33	0.49 (0.34–0.69) **
MN lymphatic and hematopoietic tissues (10)	31	0.82 (0.56–1.16)
Benign and unspecified neoplasms (11)	6	1.14 (0.42–2.49)
Diseases of the blood and blood-forming organs (12)	≤5	0.31 (0.03–1.11)
Diabetes mellitus (13)	8	0.15 (0.06–0.29) **
Mental, psychoneurotic, and personality disorders (14)	11	0.32 (0.16–0.58) **
Nervous system disorders (15)	9	0.20 (0.09–0.38) **
Heart diseases (16)	120	0.33 (0.27–0.40) **
Other diseases of the circulatory system (17)	23	0.25 (0.16–0.38) **
Diseases of the respiratory system (18)	28	0.27 (0.18–0.39) **
Diseases of the digestive system (19)	20	0.21 (0.13–0.32) **
Diseases of the skin and subcutaneous tissues (20)	≤5	0.91 (0.10–3.28)
Diseases of the musculoskeletal and connective tissue systems (21)	≤5	0.37 (0.04–1.33)
Diseases of the genito-urinary system (22)	≤5	0.08 (0.01–0.28) **
Symptoms and ill-defined conditions (23)	11	0.52 (0.26–0.93) *
Transportation injuries (24)	13	0.23 (0.12–0.40) **
Falls (25)	6	0.42 (0.15–0.91) *
Other injury (26)	41	0.45 (0.33–0.62) **
Violence (27)	26	0.30 (0.20–0.44) **
Other and unspecified (residual and blank codes) causes (28)	22	0.32 (0.20–0.48) **

* p < 0.05; ** *p* < 0.01. Abbreviations: National Institute for Occupational Safety and Health (NIOSH), 95% confidence interval (CI), malignant neoplasm (MN). ^1^ US population mortality rates from 1960–2019 were used as the reference. ^2^ Causes of death are from the NIOSH major cause of death categories and are listed with the associated NIOSH numbers in parentheses. NIOSH categories based on International Classification of Diseases, 10th revision codes [24].

**Table 3:** Observed deaths and standardized mortality ratios (SMRs) for minor cause of death categories among Fire Department of the City of New York (FDNY) rescue/recovery workers compared with US population rates ^1.^

Cause (NIOSH Major, Minor Category) ^2^	Observed	SMR (95% CI)
HIV-related (01, 03)	≤5	0.10 (0.01–0.37) **
MN esophagus (03, 08)	13	0.69 (0.36–1.17)
MN pancreas (03, 13)	20	0.71 (0.43–1.09)
MN trachea, bronchus, lung (04, 16)	34	0.29 (0.20–0.40) **
MN melanoma (09, 29)	7	0.72 (0.29–1.48)
MN mesothelioma (09, 31)	≤5	1.95 (0.52–5.00)
MN brain and other nervous (09, 33)	12	0.73 (0.38–1.28)
MN eye (09, 34)	0	0.00 (0.00–14.26)
Non-Hodgkin’s lymphoma (10, 38)	18	1.24 (0.73–1.95)
Multiple myeloma (39)	≤5	0.68 (0.22–1.59)
BN eye, brain, other nervous (11, 41)	0	0.00 (0.00–9.30)
Alcoholism (14, 49)	6	0.36 (0.13–0.78) **
Other nervous system diseases (15, 52)	9	0.21 (0.10–0.40) **
Rheumatic heart disease (16, 53)	≤5	0.82 (0.01–4.56)
Hypertension w/heart disease (16, 54)	7	0.23 (0.09–0.48) **
Ischemic heart disease (16, 55)	85	0.33 (0.27–0.41) **
Cerebrovascular disease (17, 60)	10	0.20 (0.09–0.36) **
Hypertension w/o heart disease (17, 61)	≤5	0.33 (0.09–0.84) *
Diseases of the arteries, veins, lymph nodes (17, 62)	9	0.33 (0.15–0.63) **
Pneumonia (18, 65)	≤5	0.26 (0.08–0.60) **
COPD (18, 66)	12	0.20 (0.10–0.35) **
Hernia and intestinal obstruction (19, 73)	0	0.00 (0.00–1.38)
Chronic and unspecified renal failure (22, 82)	≤5	0.06 (0.00–0.32) **
Motor vehicle-driver (24, 92)	6	0.37 (0.14–0.81) **
Motor vehicle—pedestrian (24, 94)	≤5	0.29 (0.03–1.07)
Fire in building (26, 109)	9	3.72 (1.70–7.06) **
Accidental poisoning (26, 112)	24	0.42 (0.27–0.62) **
Suicide (27, 116) ^3^	23	0.34 (0.22–0.51) **
Assault and homicide (27, 117)	≤5	0.16 (0.03–0.46) **

* *p* < 0.05; ** *p* < 0.01. Abbreviations: Fire Department of the City of New York (FDNY), National Institute for Occupational Safety and Health (NIOSH), 95% confidence interval (CI), human immunodeficiency virus (HIV), malignant neoplasm (MN), benign and unspecified neoplasms (BN), chronic obstructive pulmonary disease (COPD). ^1^ US population mortality rates from 1960–2019 were used as the reference. ^2^ Causes of death are from the NIOSH major and minor cause of death categories and are listed with the associated NIOSH numbers in parentheses. NIOSH categories based on International Classification of Diseases, 10th revision codes [24]. ^3^ LTAS category “Intentional self-harm”.

**Table 4:** Observed deaths and standardized mortality ratios (SMRs) for major causes of death among FDNY rescue/recovery workers by race compared with US population rates ^1^.

Cause (NIOSH Major Category) ^2^	Non-Hispanic White		Non-White ^3^	
	Observed	SMR (95% CI)	Observed	SMR (95% CI)
All causes	468	0.33 (0.30–0.36) **	78	0.44 (0.35–0.55) **
Tuberculosis and HIV related disease (01)	≤5	0.08 (0.00–0.44) **	≤5	0.29 (0.03–1.03)
All cancers	164	0.44 (0.38–0.52) **	27	0.66 (0.44–0.96) *
MN buccal and pharynx (02)	≤5	0.20 (0.02–0.74) **	0	0.00 (0.00–2.93)
MN digestive organs and peritoneum (03)	56	0.50 (0.38–0.65) **	14	0.99 (0.54–1.65)
MN respiratory system (04)	33	0.30 (0.20–0.41) **	≤5	0.17 (0.02–0.62) **
MN breast (05)	0	0.00 (0.00–2.85)	≤5	1.09 (0.01–6.05)
MN female genital organs (06)	0	0.00 (0.00–7.32)	≤5	2.38 (0.03–13.25)
MN male genital organs (07)	≤5	0.29 (0.09–0.68) **	≤5	0.80 (0.09–2.90)
MN urinary (08)	11	0.50 (0.25–0.90) *	0	0.00 (0.00–2.36)
MN other and unspecified sites (09)	30	0.48 (0.32–0.69) **	≤5	0.62 (0.12–1.81)
MN lymphatic and hematopoietic tissues (10)	27	0.78 (0.52–1.14)	≤5	1.16 (0.31–2.97)
Benign and unspecified neoplasms (11)	6	1.27 (0.46–2.76)	0	0.00 (0.00–7.17)
Diseases of the blood and blood-forming organs (12)	≤5	0.36 (0.04–1.31)	0	0.00 (0.00–3.78)
Diabetes mellitus (13)	≤5	0.09 (0.02–0.22) **	≤5	0.53 (0.14–1.35)
Mental, psychoneurotic, and personality disorders (14)	10	0.32 (0.16–0.60) **	≤5	0.33 (0.00–1.81)
Nervous system disorders (15)	8	0.19 (0.08–0.38) **	≤5	0.28 (0.00–1.59)
Heart diseases (16)	98	0.30 (0.25–0.37) **	22	0.53 (0.33–0.81) **
Other diseases of the circulatory system (17)	20	0.26 (0.16–0.40) **	≤5	0.21 (0.04–0.62) **
Diseases of the respiratory system (18)	28	0.29 (0.19–0.42) **	0	0.00 (0.00–0.45) **
Diseases of the digestive system (19)	19	0.22 (0.13–0.34) **	≤5	0.12 (0.00–0.69) **
Diseases of the skin and subcutaneous tissues (20)	≤5	1.06 (0.14–3.84)	0	0.00 (0.00–11.51)
Diseases of the musculoskeletal and connective tissue systems (21)	≤5	0.44 (0.05–1.57)	0	0.00 (0.00–4.47)
Diseases of the genito-urinary system (22)	≤5	0.05 (0.0–0.26) **	≤5	0.22 (0.01–1.24)
Symptoms and ill-defined conditions (23)	11	0.59 (0.29–1.05)	0	0.00 (0.00–1.42)
Transportation injuries (24)	12	0.24 (0.13–0.42) **	≤5	0.17 (0.00–0.93) *
Falls (25)	≤5	0.38 (0.12–0.88) *	≤5	1.01 (0.03–5.63)
Other injury (26)	38	0.47 (0.33–0.64) **	≤5	0.33 (0.07–0.97) *
Violence (27)	22	0.29 (0.18–0.44) **	≤5	0.38 (0.10–0.98) *
Other and unspecified (residual and blank codes) causes (28)	15	0.25 (0.14–0.41) **	7	0.81 (0.33–1.67)

* *p* < 0.05; ** *p* < 0.01. Abbreviations: Fire Department of the City of New York (FDNY), National Institute for Occupational Safety and Health (NIOSH), 95% confidence interval (CI), malignant neoplasm (MN). ^1^ US population mortality rates from 1960–2019 were used as the reference. ^2^ Causes of death are from the NIOSH major and minor cause of death categories and are listed with the associated NIOSH numbers in parentheses. NIOSH categories based on International Classification of Diseases, 10th revision codes [24]. ^3^ Non-Hispanic black, Hispanic, other.

**Table 5:** Observed deaths and standardized mortality ratios (SMRs) for major causes of death among FDNY rescue/recovery workers by work assignment compared with US population rates ^1^**^.^**

Cause (NIOSH Major Category) ^2^	Firefighters		Emergency Medical Service Providers	
	Observed	SMR	Observed	SMR
All causes	455	0.31 (0.29–0.34) **	91	0.61 (0.49–0.75) **
Tuberculosis and HIV related disease (01)	≤5	0.06 (0.0–0.36) **	≤5	0.46 (0.05–1.65)
All cancers	164	0.43 (0.37–0.51) **	27	0.81 (0.53–1.18)
MN buccal and pharynx (02)	≤5	0.20 (0.02–0.71) **	0	0.00 (0.00–3.86)
MN digestive organs and peritoneum (03)	60	0.52 (0.40–0.67) **	10	0.93 (0.45–1.72)
MN respiratory system (04)	32	0.28 (0.19–0.39) **	≤5	0.34 (0.07–0.99) *
MN breast (05)	0	0.00 (0.00–5.38)	≤5	0.65 (0.02–3.64)
MN female genital organs (06)	0	0.00 (0.00–44.02)	≤5	1.19 (0.03–6.61)
MN male genital organs (07)	6	0.33 (0.12–0.71) **	≤5	0.86 (0.02–4.79)
MN urinary (08)	10	0.45 (0.22–0.84) **	≤5	0.70 (0.02–3.88)
MN other and unspecified sites (09)	30	0.48 (0.33–0.69) **	≤5	0.59 (0.12–1.72)
MN lymphatic and hematopoietic tissues (10)	24	0.69 (0.44–1.02)	7	2.43 (0.98–5.01)
Benign and unspecified neoplasms (11)	≤5	1.04 (0.34–2.44)	≤5	2.17 (0.05–12.08)
Diseases of the blood and blood-forming organs (12)	≤5	0.17 (0.00–0.96) *	≤5	1.46 (0.04–8.14)
Diabetes mellitus (13)	≤5	0.10 (0.03–0.24) **	≤5	0.56 (0.12–1.65)
Mental, psychoneurotic, and personality disorders (14)	9	0.29 (0.24–0.36) **	≤5	0.72 (0.09–2.60)
Nervous system disorders (15)	7	0.17 (0.07–0.34) **	≤5	0.62 (0.08–2.25)
Heart diseases (16)	97	0.29 (0.24–0.36) **	23	0.74 (0.47-1.12)
Other diseases of the circulatory system (17)	18	0.22 (0.13–0.35) **	≤5	0.53 (0.17–1.24)
Diseases of the respiratory system (18)	26	0.27 (0.17–0.39) **	≤5	0.29 (0.04–1.05)
Diseases of the digestive system (19)	18	0.21 (0.12–0.33) **	≤5	0.23 (0.03–0.85) *
Diseases of the skin and subcutaneous tissues (20)	≤5	0.51 (0.01–2.84)	≤5	4.19 (0.11–23.35)
Diseases of the musculoskeletal and connective tissue systems (21)	≤5	0.42 (0.05–1.52)	0	0.00 (0.00–5.47)
Diseases of the genito-urinary system (22)	≤5	0.04 (0.00–0.24) **	≤5	0.37 (0.01–2.05)
Symptoms and ill-defined conditions (23)	9	0.47 (0.22–0.90) *	≤5	0.87 (0.11–3.15)
Transportation injuries (24)	12	0.25 (0.13–0.43) **	≤5	0.15 (0.00–0.84) *
Falls (25)	≤5	0.38 (0.12–0.88) *	≤5	0.96 (0.02–5.32)
Other injury (26)	35	0.44 (0.31–0.61) **	6	0.56 (0.20–1.21)
Violence (27)	22	0.29 (0.18–0.44) **	≤5	0.37 (0.10–0.95) *
Other and unspecified (residual and blank codes) causes (28)	17	0.27 (0.16–0.44) **	≤5	0.70 (0.23–1.64)

* *p* < 0.05; ** *p* < 0.01. Abbreviations: Fire Department of the City of New York (FDNY), National Institute for Occupational Safety and Health (NIOSH), 95% confidence interval (CI), malignant neoplasm (MN). ^1^ US population mortality rates from 1960–2019 were used as the reference. ^2^ Causes of death are from the NIOSH major and minor cause of death categories and are listed with the associated NIOSH numbers in parentheses. NIOSH categories based on International Classification of Diseases, 10th revision codes [24].

**Table 6:** Observed deaths and standardized mortality ratios (SMRs) for major causes of death among FDNY rescue/recovery workers by arrival to the World Trade Center (WTC) site compared with US population rates ^1.^

Cause (NIOSH Major Category) ^2^	High Exposure		Moderate/Low Exposure	
	Observed	SMR (95% CI)	Observed	SMR (95% CI)
All causes	86	0.41 (0.33–0.51) **	460	0.33 (0.30–0.36) **
Tuberculosis and HIV related disease (01)	≤5	0.30 (0.01–1.66)	≤5	0.12 (0.01–0.44) **
All cancers	26	0.51 (0.33–0.74) **	165	0.46 (0.39–0.53) **
MN buccal and pharynx (02)	0	0.00 (0.00–2.45)	≤5	0.21 (0.03–0.76) **
MN digestive organs and peritoneum (03)	12	0.73 (0.38–1.28)	58	0.53 (0.40–0.69) **
MN respiratory system (04)	≤5	0.20 (0.04–0.59) **	32	0.29 (0.20–0.42) **
MN breast (05)	0	0.00 (00.0–9.53)	≤5	0.55 (0.01–3.05)
MN female genital organs (06)	0	0.00 (0.00–20.23)	≤5	1.34 (0.03–7.48)
MN male genital organs (07)	≤5	1.01 (0.12–3.63)	≤5	0.28 (0.09–0.66) **
MN urinary (08)	≤5	0.72 (0.09–2.60)	9	0.44 (0.20–0.83) **
MN other and unspecified sites (09)	≤5	0.46 (0.13–1.18)	29	0.50 (0.33–0.71) **
MN lymphatic and hematopoietic tissues (10)	≤5	0.66 (0.14–1.92)	28	0.84 (0.56–1.21)
Benign and unspecified neoplasms (11)	≤5	1.50 (0.04–8.36)	≤5	1.09 (0.35–2.55)
Diseases of the blood and blood-forming organs (12)	0	0.00 (0.00–4.75)	≤5	0.35 (0.04–1.26)
Diabetes mellitus (13)	≤5	0.42 (0.09–1.24)	≤5	0.11 (0.03–0.25) **
Mental, psychoneurotic, and personality disorders (14)	0	0.00 (0.00–0.86) *	11	0.37 (0.19–0.66) **
Nervous system disorders (15)	≤5	0.20 (0.01–1.11)	8	0.20 (0.09–0.39) **
Heart diseases (16)	14	0.30 (0.16–0.51) **	106	0.34 (0.27–0.41) **
Other diseases of the circulatory system (17)	≤5	0.17 (0.02–0.63) **	21	0.27 (0.16–0.41) **
Diseases of the respiratory system (18)	≤5	0.43 (0.14–1.01)	23	0.25 (0.16–0.37) **
Diseases of the digestive system (19)	≤5	0.07 (0.00–0.41) **	19	0.23 (0.14–0.36) **
Diseases of the skin and subcutaneous tissues (20)	≤5	3.43 (0.09–19.08)	≤5	0.52 (0.01–2.92)
Diseases of the musculoskeletal and connective tissue systems (21)	≤5	1.39 (0.04–7.77)	≤5	0.21 (0.01–1.18)
Diseases of the genito-urinary system (22)	0	0.00 (0.00–1.16)	≤5	0.09 (0.01–0.32) **
Symptoms and ill-defined conditions (23)	≤5	0.67 (0.08–2.42)	9	0.49 (0.23–0.94) *
Transportation injuries (24)	≤5	0.47 (0.13–1.21)	9	0.19 (0.09–0.36) **
Falls (25)	≤5	1.11 (0.13–4.01)	≤5	0.32 (0.09–0.82) *
Other injury (26)	11	0.78 (0.39–1.40)	30	0.39 (0.27–0.56) **
Violence (27)	7	0.52 (0.21–1.08)	19	0.26 (0.16–0.40) **
Other and unspecified (residual and blank codes) causes (28)	≤5	0.42 (0.11–1.08)	18	0.30 (0.18–0.48) **

* *p* < 0.05; ** *p* < 0.01. Abbreviations: Fire Department of the City of New York (FDNY), National Institute for Occupational Safety and Health (NIOSH), 95% confidence interval (CI), malignant neoplasm (MN). ^1^ US population mortality rates from 1960–2019 were used as the reference. ^2^ Causes of death are from the NIOSH major and minor cause of death categories and are listed with the associated NIOSH numbers in parentheses. NIOSH categories based on International Classification of Diseases, 10th revision codes [24].

**Table 7:** Unadjusted and adjusted cox proportional hazards models for all-cause, cancer, heart, and respiratory disease mortality among FDNY WTC-exposed rescue/recovery workers, 9/12/2001–2017.

Variable	All-Cause Mortality				Cancer Mortality				Heart Disease Mortality				Respiratory Disease Mortality ^2^			
	HR	95% CI	AHR ^1^	95% CI	HR	95% CI	AHR ^1^	95% CI	HR	95% CI	AHR ^1^	95% CI	HR	95% CI	AHR	95% CI
WTC exposure (high vs. moderate/low)	1.03	0.82–1.30	1.24	0.98–1.58	0.88	0.58–1.32	1.15	0.75–1.78	0.78	0.45–1.37	0.93	0.52–1.68	1.25	0.47–3.28	1.44	0.49–4.23
Age on 9/11/2001 (continuous, years)			1.08	1.07–1.09			1.10	1.08–1.12			1.09	1.07–1.12			1.14	1.09–1.19
BMI (continuous, kg/m ^2^)			1.00	0.98–1.02			0.97	0.93–1.01			1.03	0.99–1.07			1.01	0.92–1.10
Race (non-Hispanic white vs. non-white) ^3^			0.84	0.64–1.12			0.75	0.47–1.19			0.71	0.39–1.31			N/A	N/A
Work assignment (firefighter vs. EMS)			0.51	0.38–0.69			0.58	0.33–1.02			0.47	0.25–0.88			0.79	0.15–4.03
Sex (male vs. female)			1.52	0.84–2.74			1.05	0.40–2.77			1.65	0.46–5.89			0.34	0.03–3.50
Smoking (ever vs. never)			1.54	1.27–1.86			1.73	1.24–2.42			1.89	1.22–2.93			0.90	0.38–2.12

Abbreviations: Fire Department of the City of New York (FDNY), World Trade Center (WTC), adjusted hazard ratio (AHR), body mass index (BMI), emergency medical service providers (EMS). ^1^ The total N value was 15,058 due to missing covariates. ^2^ Race restricted to non-Hispanic whites as all 28 deaths from respiratory disease occurred among that category. The total N value of AHR models was 13,114 due to missing covariates. ^3^ Non-Hispanic black, Hispanic, other.
