# Correction: Colbeth et al. Mortality among Fire Department of the City of New York Rescue and Recovery Workers Exposed to the World Trade Center Disaster, 2001–2017. *Int. J. Environ. Res. Public Health* 2020, *17*, 6266

**DOI:** 10.3390/ijerph20166585

**Published:** 2023-08-16

**Authors:** Hilary L. Colbeth, Rachel Zeig-Owens, Charles B. Hall, Mayris P. Webber, Theresa M. Schwartz, David J. Prezant

**Affiliations:** 1 Fire Department of the City of New York, Bureau of Health Services, 9 Metrotech Center, Brooklyn, NY 11201, USA; 2 Department of Medicine, Pulmonology Division, Montefiore Medical Center, Bronx, NY 10467, USA; 3 Department of Epidemiology and Population Health, Albert Einstein College of Medicine, Bronx, NY 10461, USA; 4 Department of Medicine, Pulmonology Division, Albert Einstein College of Medicine, Bronx, NY 10461, USA

The original publication [1] contained the following errors in need of correction:

## Text Correction

We found an error in the Life Table Analysis System (LTAS) project used to estimate expected numbers of deaths in our study population. Expected deaths should have been calculated using person-time at risk beginning from the study start date, and instead were calculated using participants’ person-time at risk starting from 1 January 1960 or their date of birth, whichever came later. Expected death counts were therefore inflated, resulting in Standardized Mortality Ratios (SMRs) for all-cause and cause specific mortality of the original publication were slightly underestimated due to the inclusion of excess person-time in the LTAS function used to estimate them.

Corrections (in bold) have been made to the following sections:

## Abstract

“The World Trade Center (WTC) attacks on 11 September 2001 have consistently been associated with elevated rates of physical and mental health morbidities, while evidence about mortality has been limited. We examined mortality between 12 September 2001 and 31 December 2017 among 15,431 WTC-exposed Fire Department of the City of New York (FDNY) firefighters and emergency medical service providers (EMS), specifically assessing associations between intensity of WTC-exposure and mortality risk. Standardized mortality ratios (SMR) and 95% confidence intervals (CI) compared FDNY cohort mortality with the US general population using life table analysis. Deaths were identified via linkage to the National Death Index. Cox proportional hazards regression models were used to identify associations between intensity of WTC exposure and mortality, accounting for age, sex, race/ethnicity, smoking history, and other relevant confounders. We identified 546 deaths and a lower than expected all-cause mortality rate (**SMR = 0.34; 95% CI, 0.31–0.37**). No cause-specific SMRs were meaningfully elevated. Mortality hazard ratios showed no association or linear trend with level of WTC exposure. Our results provide evidence of the healthy worker effect, despite exposure to the World Trade Center. More follow-up time may be needed to assess the full impact of WTC exposure on mortality in this occupational population.”

## In Section 2.3. Statistical Analysis, paragraph 1

“Demographic and other characteristics of the study population were assessed as proportions, medians, interquartile ranges, and means (SDs), as appropriate. The observation period began on 12 September 2001 and follow-up ended at death or the study end date (31 December 2017), whichever was earlier. **The National Institute for Occupational Safety (NIOSH) Life Table Analysis System (LTAS.NET)** [25,26] **was used to calculate standardized mortality ratios with the US general population as the referent (1960–2019), and RStudio version 4.1 was used to check the LTAS output.** The underlying cause of death was categorized per LTAS’s 119-cause rate file [25] **which uses ICD-10 codes mapped to 119 minor and 28 major cause of death categories** [24]. For each rescue/recovery worker, observed person-years-at-risk were stratified by sex (male, female), race (non-Hispanic white, non-white, non-Hispanic black, Hispanic, and other), and five-year bands of age and calendar period. Yearly expected mortality was calculated by multiplying the stratum’s person-years-at-risk by the corresponding cause-specific US population mortality rate [25]. The expected numbers were summed across strata to obtain cause-specific and total expected number of deaths. SMRs were estimated as the ratio of the observed to the expected deaths. Ninety-five percent confidence intervals (CI) were estimated based on a Poisson distribution for the observed outcome, with exact limits for outcomes with 10 or fewer occurrences [27].”

## In Section 3.2. Mortality Among the Full Cohort, paragraph 1

“Compared to the US population referent, all-cause mortality was significantly lower than expected **(SMR = 0.34; 95% CI, 0.31–0.37)** (Table 2). Similarly, SMRs for many of the major cause-of-death categories, including all cancers **(SMR = 0.46; 95% CI, 0.40–0.54), diseases of the heart (SMR = 0.33; 95% CI, 0.27–0.40), other diseases of the circulatory system (SMR = 0.25; 95% CI, 0.16–0.38), and diseases of the respiratory system (SMR = 0.27; 95% CI, 0.18–0.39), were significantly lower than expected. Among cancer deaths, those with the most observed cases demonstrated significantly lower than expected mortality: digestive organs and peritoneum (SMR = 0.56; 95% CI, 0.43–0.70), respiratory system (SMR = 0.28; 95% CI, 0.20–0.39), and other and unspecified sites (SMR = 0.49; 95% CI, 0.34–0.69).** Observed deaths and SMRs among NIOSH minor cause of death categories, such as suicide, are presented in Table 3. **None were significantly elevated in the overall population, except for mortality caused by building fires (SMR = 3.72; 95% CI, 1.70–7.06)**.”

## In Section 3.3. Race, Work Assignment, and WTC-Exposure

“All-cause mortality among non-Hispanic whites **(SMR = 0.33; 95% CI, 0.30–0.36) and non-whites (SMR = 0.44; 95% CI, 0.35–0.55)** was similar and significantly below the US general population (Table 4). Cancers were the leading cause of death among non-Hispanic whites and non-whites **(SMR = 0.44; 95% CI, 0.38–0.52, n = 164 and SMR = 0.66; 95% CI, 0.44–0.96, n = 27),** resulting in approximately 35% of deaths in each respective group. Cancer deaths among non-whites were closely followed in number by heart disease mortality **(SMR = 0.53; 95% CI, 0.33–0.81; n = 22).** All respiratory disease deaths occurred among non-Hispanic whites **(SMR = 0.29; 95% CI, 0.19–0.42; n = 28).**

Table 5 shows that firefighters were characterized by decreased **all-cause mortality (SMR = 0.31; 95% CI, 0.29–0.34), all-cancer mortality (SMR = 0.43; 95% CI, 0.37–0.51), heart disease mortality (SMR = 0.29; 95% CI, 0.24–0.36) and respiratory disease mortality (SMR = 0.27; 95% CI, 0.17–0.39).** Firefighters also showed significant decreases compared with the US general population from mental-health-related causes of death, such as suicide **(SMR = 0.31; 95% CI, 0.19–0.49; n = 19) and alcoholism (SMR = 0.33; 95% CI, 0.11–0.77; n ≤ 5) (data not shown). Only death from building fires showed an excess (SMR = 4.26; 95% CI, 1.94–8.09; n = 9)** (data not shown); all of these deaths occurred in firefighters who were on active duty (i.e., during work). Among EMS, results in major cause of death categories were similar to those of firefighters. 

Those who arrived the morning of 11 September 2001 (high WTC exposure) demonstrated significantly lower than expected all-cause mortality **(SMR = 0.41; 95% CI, 0.33–0.51), all-cancer mortality (SMR = 0.51; 95% CI, 0.33–0.74), and heart disease mortality (SMR = 0.30; 95% CI, 0.16–0.51)** (Table 6). No major causes of death were significantly elevated. Results were similar among those with moderate/low WTC exposure (arrived the afternoon of 11 September 2001–25 July 2002).”

## In Section 4. Discussion, paragraphs 3 and 5

“With some exceptions, our results are consistent with SMRs previously reported by the WTC Health Registry and the WTC Health Program General Responder Cohort. Our all-cause mortality ratio **(SMR = 0.34; 95% CI, 0.31–0.37)** was lower than both the rescue/recovery workers of the WTC Health Registry [17] (SMR = 0.69, 95% CI, 0.65–0.74) and the General Responder Cohort [18] (SMR = 0.43; 95% CI, 0.39–0.48). In contrast with the WTC Health Registry rescue/recovery workers, the FDNY cohort did not experience higher than expected mortality rates, even among the minor cause categories which included malignant cancers, such as pancreatic or non-Hodgkin’s lymphoma, nervous system diseases, and suicide. In addition, our Cox proportional hazards model results differed from the WTC Health Registry, which found significant associations between higher levels of WTC exposure and all-cause mortality among rescue/recovery workers. These differences may be partially explained by the WTC Health Registry rescue/recovery cohort being composed mostly of police, construction, and communication workers who did not receive pre-hire and annual medical exams prior to 11 September 2001 and therefore may not be influenced by the healthy worker effect to the same degree as the FDNY cohort [17]. Regarding the WTC-exposed General Responder Cohort, FDNY cause-specific mortality results were similar, with the exception of neoplasms of lymphatic and hematopoietic tissue and benign and unspecified neoplasms, which they found to be slightly elevated. The General Responder Cohort also reflected the results of our Cox proportional hazards model in their report of an increased, albeit non-significant, risk of all-cause mortality among those directly in the dust cloud (AHR = 1.13; 95% CI, 0.81–1.59), an equivalent measure to our high exposure category, and likewise did not identify an exposure-response gradient (p-trend = 0.88). 

Expected associations for well-established risk factors of premature mortality in our cohort provide confidence in our results. For example, SMRs of the cause-specific minor categories were overwhelmingly lower than expected compared with the general population, **with the exception of elevated rates of death from a building fire (SMR = 3.72), which is not surprising given that our population was nearly 89% firefighters, and, though not significantly, mesothelioma (SMR = 2.05),** which has been reported as elevated among firefighters compared with the general population [28,38]. Secondly, as was emphasized through SMR stratification by WTC exposure, our Cox proportional hazards analyses demonstrated that WTC exposure intensity was not related to mortality risk while more typical risk factors such as being male, cigarette smoking, and older age conferred increased risk. Of the known correlates, the strongest association was with smoking history, in which ever-smokers demonstrated 50% or greater increased risk of death from all-causes, cancer, and heart disease compared with never-smokers. Ever smoking was not associated with respiratory disease mortality, likely due to a lack of statistical power with only 28 deaths due to respiratory disease.”

## Error in Table

Table 2, Table 3, Table 4, Table 5 and Table 6 in the original publication have been corrected below:

**Table 2:** Observed deaths and standardized mortality ratios (SMRs) for major cause of death categories among World Trade Center-exposed Fire Department rescue/recovery workers of the City of New York, 12 September 2001–31 December 2017, compared with US population rates ^1.^

Cause (National Institute for Occupational Safety (NIOSH) Major Category) ^2^	Observed	SMR (95% CI)
All causes	546	0.34 (0.31–0.37) **
Tuberculosis and HIV related disease (01)	≤5	0.15 (0.03–0.44) **
All cancers	191	0.46 (0.40–0.54) **
MN buccal and pharynx (02)	≤5	0.18 (0.02–0.65) **
MN digestive organs and peritoneum (03)	70	0.56 (0.43–0.70) **
MN respiratory system (04)	35	0.28 (0.20–0.39) **
MN breast (05)	≤5	0.45 (0.01–2.51)
MN female genital organs (06)	≤5	1.09 (0.01–6.05)
MN male genital organs (07)	7	0.36 (0.14–0.74) **
MN urinary (08)	11	0.47 (0.23–0.84) **
MN other and unspecified sites (09)	33	0.49 (0.34–0.69) **
MN lymphatic and hematopoietic tissues (10)	31	0.82 (0.56–1.16)
Benign and unspecified neoplasms (11)	6	1.14 (0.42–2.49)
Diseases of the blood and blood-forming organs (12)	≤5	0.31 (0.03–1.11)
Diabetes mellitus (13)	8	0.15 (0.06–0.29) **
Mental, psychoneurotic, and personality disorders (14)	11	0.32 (0.16–0.58) **
Nervous system disorders (15)	9	0.20 (0.09–0.38) **
Heart diseases (16)	120	0.33 (0.27–0.40) **
Other diseases of the circulatory system (17)	23	0.25 (0.16–0.38) **
Diseases of the respiratory system (18)	28	0.27 (0.18–0.39) **
Diseases of the digestive system (19)	20	0.21 (0.13–0.32) **
Diseases of the skin and subcutaneous tissues (20)	≤5	0.91 (0.10–3.28)
Diseases of the musculoskeletal and connective tissue systems (21)	≤5	0.37 (0.04–1.33)
Diseases of the genito-urinary system (22)	≤5	0.08 (0.01–0.28) **
Symptoms and ill-defined conditions (23)	11	0.52 (0.26–0.93) *
Transportation injuries (24)	13	0.23 (0.12–0.40) **
Falls (25)	6	0.42 (0.15–0.91) *
Other injury (26)	41	0.45 (0.33–0.62) **
Violence (27)	26	0.30 (0.20–0.44) **
Other and unspecified (residual and blank codes) causes (28)	22	0.32 (0.20–0.48) **

* p < 0.05; ** *p* < 0.01. Abbreviations: National Institute for Occupational Safety and Health (NIOSH), 95% confidence interval (CI), malignant neoplasm (MN). ^1^ US population mortality rates from 1960–2019 were used as the reference. ^2^ Causes of death are from the NIOSH major cause of death categories and are listed with the associated NIOSH numbers in parentheses. NIOSH categories based on International Classification of Diseases, 10th revision codes [24].

**Table 3:** Observed deaths and standardized mortality ratios (SMRs) for minor cause of death categories among Fire Department of the City of New York (FDNY) rescue/recovery workers compared with US population rates ^1.^

Cause (NIOSH Major, Minor Category) ^2^	Observed	SMR (95% CI)
HIV-related (01, 03)	≤5	0.10 (0.01–0.37) **
MN esophagus (03, 08)	13	0.69 (0.36–1.17)
MN pancreas (03, 13)	20	0.71 (0.43–1.09)
MN trachea, bronchus, lung (04, 16)	34	0.29 (0.20–0.40) **
MN melanoma (09, 29)	7	0.72 (0.29–1.48)
MN mesothelioma (09, 31)	≤5	1.95 (0.52–5.00)
MN brain and other nervous (09, 33)	12	0.73 (0.38–1.28)
MN eye (09, 34)	0	0.00 (0.00–14.26)
Non-Hodgkin’s lymphoma (10, 38)	18	1.24 (0.73–1.95)
Multiple myeloma (39)	≤5	0.68 (0.22–1.59)
BN eye, brain, other nervous (11, 41)	0	0.00 (0.00–9.30)
Alcoholism (14, 49)	6	0.36 (0.13–0.78) **
Other nervous system diseases (15, 52)	9	0.21 (0.10–0.40) **
Rheumatic heart disease (16, 53)	≤5	0.82 (0.01–4.56)
Hypertension w/heart disease (16, 54)	7	0.23 (0.09–0.48) **
Ischemic heart disease (16, 55)	85	0.33 (0.27–0.41) **
Cerebrovascular disease (17, 60)	10	0.20 (0.09–0.36) **
Hypertension w/o heart disease (17, 61)	≤5	0.33 (0.09–0.84) *
Diseases of the arteries, veins, lymph nodes (17, 62)	9	0.33 (0.15–0.63) **
Pneumonia (18, 65)	≤5	0.26 (0.08–0.60) **
COPD (18, 66)	12	0.20 (0.10–0.35) **
Hernia and intestinal obstruction (19, 73)	0	0.00 (0.00–1.38)
Chronic and unspecified renal failure (22, 82)	≤5	0.06 (0.00–0.32) **
Motor vehicle-driver (24, 92)	6	0.37 (0.14–0.81) **
Motor vehicle—pedestrian (24, 94)	≤5	0.29 (0.03–1.07)
Fire in building (26, 109)	9	3.72 (1.70–7.06) **
Accidental poisoning (26, 112)	24	0.42 (0.27–0.62) **
Suicide (27, 116) ^3^	23	0.34 (0.22–0.51) **
Assault and homicide (27, 117)	≤5	0.16 (0.03–0.46) **

* *p* < 0.05; ** *p* < 0.01. Abbreviations: Fire Department of the City of New York (FDNY), National Institute for Occupational Safety and Health (NIOSH), 95% confidence interval (CI), human immunodeficiency virus (HIV), malignant neoplasm (MN), benign and unspecified neoplasms (BN), chronic obstructive pulmonary disease (COPD). ^1^ US population mortality rates from 1960–2019 were used as the reference. ^2^ Causes of death are from the NIOSH major and minor cause of death categories and are listed with the associated NIOSH numbers in parentheses. NIOSH categories based on International Classification of Diseases, 10th revision codes [24]. ^3^ LTAS category “Intentional self-harm.”

**Table 4:** Observed deaths and standardized mortality ratios (SMRs) for major causes of death among FDNY rescue/recovery workers by race compared with US population rates ^1^.

Cause (NIOSH Major Category) ^2^	Non-Hispanic White		Non-White ^3^	
	Observed	SMR (95% CI)	Observed	SMR (95% CI)
All causes	468	0.33 (0.30–0.36) **	78	0.44 (0.35–0.55) **
Tuberculosis and HIV related disease (01)	≤5	0.08 (0.00–0.44) **	≤5	0.29 (0.03–1.03)
All cancers	164	0.44 (0.38–0.52) **	27	0.66 (0.44–0.96) *
MN buccal and pharynx (02)	≤5	0.20 (0.02–0.74) **	0	0.00 (0.00–2.93)
MN digestive organs and peritoneum (03)	56	0.50 (0.38–0.65) **	14	0.99 (0.54–1.65)
MN respiratory system (04)	33	0.30 (0.20–0.41) **	≤5	0.17 (0.02–0.62) **
MN breast (05)	0	0.00 (0.00–2.85)	≤5	1.09 (0.01–6.05)
MN female genital organs (06)	0	0.00 (0.00–7.32)	≤5	2.38 (0.03–13.25)
MN male genital organs (07)	≤5	0.29 (0.09–0.68) **	≤5	0.80 (0.09–2.90)
MN urinary (08)	11	0.50 (0.25–0.90) *	0	0.00 (0.00–2.36)
MN other and unspecified sites (09)	30	0.48 (0.32–0.69) **	≤5	0.62 (0.12–1.81)
MN lymphatic and hematopoietic tissues (10)	27	0.78 (0.52–1.14)	≤5	1.16 (0.31–2.97)
Benign and unspecified neoplasms (11)	6	1.27 (0.46–2.76)	0	0.00 (0.00–7.17)
Diseases of the blood and blood-forming organs (12)	≤5	0.36 (0.04–1.31)	0	0.00 (0.00–3.78)
Diabetes mellitus (13)	≤5	0.09 (0.02–0.22) **	≤5	0.53 (0.14–1.35)
Mental, psychoneurotic, and personality disorders (14)	10	0.32 (0.16–0.60) **	≤5	0.33 (0.00–1.81)
Nervous system disorders (15)	8	0.19 (0.08–0.38) **	≤5	0.28 (0.00–1.59)
Heart diseases (16)	98	0.30 (0.25–0.37) **	22	0.53 (0.33–0.81) **
Other diseases of the circulatory system (17)	20	0.26 (0.16–0.40) **	≤5	0.21 (0.04–0.62) **
Diseases of the respiratory system (18)	28	0.29 (0.19–0.42) **	0	0.00 (0.00–0.45) **
Diseases of the digestive system (19)	19	0.22 (0.13–0.34) **	≤5	0.12 (0.00–0.69) **
Diseases of the skin and subcutaneous tissues (20)	≤5	1.06 (0.14–3.84)	0	0.00 (0.00–11.51)
Diseases of the musculoskeletal and connective tissue systems (21)	≤5	0.44 (0.05–1.57)	0	0.00 (0.00–4.47)
Diseases of the genito-urinary system (22)	≤5	0.05 (0.0–0.26) **	≤5	0.22 (0.01–1.24)
Symptoms and ill-defined conditions (23)	11	0.59 (0.29–1.05)	0	0.00 (0.00–1.42)
Transportation injuries (24)	12	0.24 (0.13–0.42) **	≤5	0.17 (0.00–0.93) *
Falls (25)	≤5	0.38 (0.12–0.88) *	≤5	1.01 (0.03–5.63)
Other injury (26)	38	0.47 (0.33–0.64) **	≤5	0.33 (0.07–0.97) *
Violence (27)	22	0.29 (0.18–0.44) **	≤5	0.38 (0.10–0.98) *
Other and unspecified (residual and blank codes) causes (28)	15	0.25 (0.14–0.41) **	7	0.81 (0.33–1.67)

* *p* < 0.05; ** *p* < 0.01. Abbreviations: Fire Department of the City of New York (FDNY), National Institute for Occupational Safety and Health (NIOSH), 95% confidence interval (CI), malignant neoplasm (MN). ^1^ US population mortality rates from 1960–2019 were used as the reference. ^2^ Causes of death are from the NIOSH major and minor cause of death categories and are listed with the associated NIOSH numbers in parentheses. NIOSH categories based on International Classification of Diseases, 10th revision codes [24]. ^3^ Non-Hispanic black, Hispanic, other.

**Table 5:** Observed deaths and standardized mortality ratios (SMRs) for major causes of death among FDNY rescue/recovery workers by work assignment compared with US population rates ^1^**^.^**

Cause (NIOSH Major Category) ^2^	Firefighters		Emergency Medical Service Providers	
	Observed	SMR	Observed	SMR
All causes	455	0.31 (0.29–0.34) **	91	0.61 (0.49–0.75) **
Tuberculosis and HIV related disease (01)	≤5	0.06 (0.0–0.36) **	≤5	0.46 (0.05–1.65)
All cancers	164	0.43 (0.37–0.51) **	27	0.81 (0.53–1.18)
MN buccal and pharynx (02)	≤5	0.20 (0.02–0.71) **	0	0.00 (0.00–3.86)
MN digestive organs and peritoneum (03)	60	0.52 (0.40–0.67) **	10	0.93 (0.45–1.72)
MN respiratory system (04)	32	0.28 (0.19–0.39) **	≤5	0.34 (0.07–0.99) *
MN breast (05)	0	0.00 (0.00–5.38)	≤5	0.65 (0.02–3.64)
MN female genital organs (06)	0	0.00 (0.00–44.02)	≤5	1.19 (0.03–6.61)
MN male genital organs (07)	6	0.33 (0.12–0.71) **	≤5	0.86 (0.02–4.79)
MN urinary (08)	10	0.45 (0.22–0.84) **	≤5	0.70 (0.02–3.88)
MN other and unspecified sites (09)	30	0.48 (0.33–0.69) **	≤5	0.59 (0.12–1.72)
MN lymphatic and hematopoietic tissues (10)	24	0.69 (0.44–1.02)	7	2.43 (0.98–5.01)
Benign and unspecified neoplasms (11)	≤5	1.04 (0.34–2.44)	≤5	2.17 (0.05–12.08)
Diseases of the blood and blood-forming organs (12)	≤5	0.17 (0.00–0.96) *	≤5	1.46 (0.04–8.14)
Diabetes mellitus (13)	≤5	0.10 (0.03–0.24) **	≤5	0.56 (0.12–1.65)
Mental, psychoneurotic, and personality disorders (14)	9	0.29 (0.24–0.36) **	≤5	0.72 (0.09–2.60)
Nervous system disorders (15)	7	0.17 (0.07–0.34) **	≤5	0.62 (0.08–2.25)
Heart diseases (16)	97	0.29 (0.24–0.36) **	23	0.74 (0.47-1.12)
Other diseases of the circulatory system (17)	18	0.22 (0.13–0.35) **	≤5	0.53 (0.17–1.24)
Diseases of the respiratory system (18)	26	0.27 (0.17–0.39) **	≤5	0.29 (0.04–1.05)
Diseases of the digestive system (19)	18	0.21 (0.12–0.33) **	≤5	0.23 (0.03–0.85) *
Diseases of the skin and subcutaneous tissues (20)	≤5	0.51 (0.01–2.84)	≤5	4.19 (0.11–23.35)
Diseases of the musculoskeletal and connective tissue systems (21)	≤5	0.42 (0.05–1.52)	0	0.00 (0.00–5.47)
Diseases of the genito-urinary system (22)	≤5	0.04 (0.00–0.24) **	≤5	0.37 (0.01–2.05)
Symptoms and ill-defined conditions (23)	9	0.47 (0.22–0.90) *	≤5	0.87 (0.11–3.15)
Transportation injuries (24)	12	0.25 (0.13–0.43) **	≤5	0.15 (0.00–0.84) *
Falls (25)	≤5	0.38 (0.12–0.88) *	≤5	0.96 (0.02–5.32)
Other injury (26)	35	0.44 (0.31–0.61) **	6	0.56 (0.20–1.21)
Violence (27)	22	0.29 (0.18–0.44) **	≤5	0.37 (0.10–0.95) *
Other and unspecified (residual and blank codes) causes (28)	17	0.27 (0.16–0.44) **	≤5	0.70 (0.23–1.64)

* *p* < 0.05; ** *p* < 0.01. Abbreviations: Fire Department of the City of New York (FDNY), National Institute for Occupational Safety and Health (NIOSH), 95% confidence interval (CI), malignant neoplasm (MN). ^1^ US population mortality rates from 1960–2019 were used as the reference. ^2^ Causes of death are from the NIOSH major and minor cause of death categories and are listed with the associated NIOSH numbers in parentheses. NIOSH categories based on International Classification of Diseases, 10th revision codes [24].

**Table 6:** Observed deaths and standardized mortality ratios (SMRs) for major causes of death among FDNY rescue/recovery workers by arrival to the World Trade Center (WTC) site compared with US population rates ^1.^

Cause (NIOSH Major Category) ^2^	High Exposure		Moderate/Low Exposure	
	Observed	SMR (95% CI)	Observed	SMR (95% CI)
All causes	86	0.41 (0.33–0.51) **	460	0.33 (0.30–0.36) **
Tuberculosis and HIV related disease (01)	≤5	0.30 (0.01–1.66)	≤5	0.12 (0.01–0.44) **
All cancers	26	0.51 (0.33–0.74) **	165	0.46 (0.39–0.53) **
MN buccal and pharynx (02)	0	0.00 (0.00–2.45)	≤5	0.21 (0.03–0.76) **
MN digestive organs and peritoneum (03)	12	0.73 (0.38–1.28)	58	0.53 (0.40–0.69) **
MN respiratory system (04)	≤5	0.20 (0.04–0.59) **	32	0.29 (0.20–0.42) **
MN breast (05)	0	0.00 (00.0–9.53)	≤5	0.55 (0.01–3.05)
MN female genital organs (06)	0	0.00 (0.00–20.23)	≤5	1.34 (0.03–7.48)
MN male genital organs (07)	≤5	1.01 (0.12–3.63)	≤5	0.28 (0.09–0.66) **
MN urinary (08)	≤5	0.72 (0.09–2.60)	9	0.44 (0.20–0.83) **
MN other and unspecified sites (09)	≤5	0.46 (0.13–1.18)	29	0.50 (0.33–0.71) **
MN lymphatic and hematopoietic tissues (10)	≤5	0.66 (0.14–1.92)	28	0.84 (0.56–1.21)
Benign and unspecified neoplasms (11)	≤5	1.50 (0.04–8.36)	≤5	1.09 (0.35–2.55)
Diseases of the blood and blood-forming organs (12)	0	0.00 (0.00–4.75)	≤5	0.35 (0.04–1.26)
Diabetes mellitus (13)	≤5	0.42 (0.09–1.24)	≤5	0.11 (0.03–0.25) **
Mental, psychoneurotic, and personality disorders (14)	0	0.00 (0.00–0.86) *	11	0.37 (0.19–0.66) **
Nervous system disorders (15)	≤5	0.20 (0.01–1.11)	8	0.20 (0.09–0.39) **
Heart diseases (16)	14	0.30 (0.16–0.51) **	106	0.34 (0.27–0.41) **
Other diseases of the circulatory system (17)	≤5	0.17 (0.02–0.63) **	21	0.27 (0.16–0.41) **
Diseases of the respiratory system (18)	≤5	0.43 (0.14–1.01)	23	0.25 (0.16–0.37) **
Diseases of the digestive system (19)	≤5	0.07 (0.00–0.41) **	19	0.23 (0.14–0.36) **
Diseases of the skin and subcutaneous tissues (20)	≤5	3.43 (0.09–19.08)	≤5	0.52 (0.01–2.92)
Diseases of the musculoskeletal and connective tissue systems (21)	≤5	1.39 (0.04–7.77)	≤5	0.21 (0.01–1.18)
Diseases of the genito-urinary system (22)	0	0.00 (0.00–1.16)	≤5	0.09 (0.01–0.32) **
Symptoms and ill-defined conditions (23)	≤5	0.67 (0.08–2.42)	9	0.49 (0.23–0.94) *
Transportation injuries (24)	≤5	0.47 (0.13–1.21)	9	0.19 (0.09–0.36) **
Falls (25)	≤5	1.11 (0.13–4.01)	≤5	0.32 (0.09–0.82) *
Other injury (26)	11	0.78 (0.39–1.40)	30	0.39 (0.27–0.56) **
Violence (27)	7	0.52 (0.21–1.08)	19	0.26 (0.16–0.40) **
Other and unspecified (residual and blank codes) causes (28)	≤5	0.42 (0.11–1.08)	18	0.30 (0.18–0.48) **

* *p* < 0.05; ** *p* < 0.01. Abbreviations: Fire Department of the City of New York (FDNY), National Institute for Occupational Safety and Health (NIOSH), 95% confidence interval (CI), malignant neoplasm (MN). ^1^ US population mortality rates from 1960–2019 were used as the reference. ^2^ Causes of death are from the NIOSH major and minor cause of death categories and are listed with the associated NIOSH numbers in parentheses. NIOSH categories based on International Classification of Diseases, 10th revision codes [24].

The authors apologize for any inconvenience caused to readers. The scientific conclusions of this article remain the same.
